# HACD3 Prevents PB1 from Autophagic Degradation to Facilitate the Replication of Influenza A Virus

**DOI:** 10.3390/v16050702

**Published:** 2024-04-29

**Authors:** Qibing Li, Li Jiang, Yihan Wang, Xuwei Liu, Bo Wang, Zhibo Shan, Yi-Han Wang, Yuqin Wang, Hualan Chen, Chengjun Li

**Affiliations:** State Key Laboratory for Animal Disease Control and Prevention, Harbin Veterinary Research Institute, Chinese Academy of Agricultural Sciences, Harbin 150069, China; qlibevis@163.com (Q.L.); jiangli@caas.cn (L.J.); wangyihan0825@163.com (Y.W.); jlxuwei11@163.com (X.L.); wangbocaas@163.com (B.W.); szb1225@163.com (Z.S.); wangyihan741@126.com (Y.-H.W.); yuckywon@163.com (Y.W.)

**Keywords:** HACD3, influenza A virus, replication, PB1, autophagy

## Abstract

Influenza A virus (IAV) continues to pose serious threats to the global animal industry and public health security. Identification of critical host factors engaged in the life cycle of IAV and elucidation of the underlying mechanisms of their action are particularly important for the discovery of potential new targets for the development of anti-influenza drugs. Herein, we identified Hydroxyacyl-CoA Dehydratase 3 (HACD3) as a new host factor that supports the replication of IAV. Downregulating the expression of HACD3 reduced the level of viral PB1 protein in IAV-infected cells and in cells that were transiently transfected to express PB1. Silencing HACD3 expression had no effect on the level of *PB1* mRNA but could promote the lysosome-mediated autophagic degradation of PB1 protein. Further investigation revealed that HACD3 interacted with PB1 and selective autophagic receptor SQSTM1/p62, and HACD3 competed with SQSTM1/p62 for the interaction with PB1, which prevented PB1 from SQSTM1/p62-mediated autophagic degradation. Collectively, these findings establish that HACD3 plays a positive regulatory role in IAV replication by stabilizing the viral PB1 protein.

## 1. Introduction

The influenza virus, a member of the family Orthomyxoviridae, is divided into four types, A, B, C, and D, on the basis of the antigenic differences of the viral NP and M1 proteins [1]. The influenza A virus (IAV) is the causative agent for seasonal influenza epidemics and occasional pandemics. IAV is an enveloped virus containing an eight-segmented negative-sense RNA genome. Ten major proteins and as many as eight accessory proteins are encoded by the IAV genome [2]. The viral genomic RNA (vRNA) is encapsidated by viral nucleoproteins (NPs) and the three viral RNA-dependent RNA polymerase (RdRp) subunits [i.e., polymerase basic protein 1 (PB1), polymerase basic protein 2 (PB2), and polymerase acidic protein (PA)] to form the viral ribonucleoprotein (vRNP) complex, which is responsible for catalyzing the transcription and replication of the viral genome [3,4,5]. PB1 is the catalytic core of the RdRp of IAV, which binds to the terminus of viral RNA to initiate the transcription and replication of viral genome [6,7]. It has been previously demonstrated that SUMOylation at position 612 is critical for PB1 to maintain its vRNA-binding ability, which plays an important role in the pathogenesis and transmission of IAV [8]. In addition, several other mutations of PB1, e.g., Y436H, S524G, and D622G [9,10,11], have also been implicated in the pathogenicity of IAV. 

The autophagy pathway is recognized as a component of the host defense system, which plays important roles in maintaining cellular homeostasis through lysosomal degradation of protein aggregates and aged or damaged organelles. Growing evidence indicates that autophagy machinery is triggered by infections of various viruses [12,13]. In particular, the autophagy pathway is also involved in the regulation of IAV replication [14,15,16]. The formation of autophagosomes is induced upon IAV infection, as reflected by the enhanced expression of the autophagosomal marker microtubule-associated protein light chain 3-II (LC3-II) [16]. The HA protein of IAV binds to HSP90AA1, which inhibits the AKT/mTOR signaling pathway, thereby activating autophagy reactions [17]. The interaction between NS1 of the 2009 pandemic H1N1 virus and LRPPC disrupts the LRPPC-Beclin1 complex, thereby inducing autophagy and promoting virus replication [18]. The NP and M2 proteins of IAV can induce autophagy through regulating the AKT-mTOR-dependent signaling pathway and the expression of HSP90AA1, resulting in enhanced replication of IAV [15,17]. The PB1 and NP proteins interact with MAVS and the autophagy receptor NBR1 or substrate receptor protein TOLLIP, and mediate selective autophagy or mitophagy to degrade MAVS, thereby blocking MAVS-mediated antiviral immunity and promoting viral replication [19,20].

HACD3 (Hydroxyacyl-CoA Dehydratase 3, HACD3), also known as BIND1 or PTPLAD1, consists of 363 amino acids. It belongs to the set of butyrate-induced genes [21] and is involved in the fatty acid biosynthetic process that is attributed to its 3-hydroxy acyl-CoA dehydratase activity [22,23]. HACD3 is a component of the Rac1 signaling pathway [24], and is also important for insulin receptor internalization [25]. HACD3 plays a crucial role in the replication of the hepatitis C virus (HCV) by interacting with the viral NS5A protein [26]. However, it remains unclear whether HACD3 engages in the replication cycle of IAV.

## 2. Materials and Methods

### 2.1. Cells and Virus 

Human lung carcinoma cells (A549, ATCC CCL-185), human embryonic kidney cells (HEK293T, ATCC CRL-3216), and Madin-Darby canine kidney (MDCK) cells were cultured as described previously [27]. 

The A/WSN/33 (WSN, H1N1) strain of IAV was propagated in MDCK cells as described previously [27]. 

### 2.2. Plasmids

The amplification of the *HACD3* gene was performed from total cellular mRNAs of A549 cells by reverse-transcription PCR with Superscript III reverse transcriptase (Invitrogen, Carlsbad, CA, USA). The open reading frame (ORF) of the amplified *HACD3* gene was subsequently cloned into the pCAGGS expression vector with or without a C-terminal V5 or Myc-tag. The full-length form (amino acids 1–362) and truncated mutants (amino acids 1–149, 150–256, and 257–362) of *HACD3* were cloned into pCAGGS with a N-terminal GST-tag. The ORFs of the *OPTN*, *NDP52*, *SQSTM1/p62*, *TAX1BP1*, and *NBR1* genes that were amplified from total cellular mRNAs of A549 cells were also cloned into pCAGGS with a C-terminal V5 tag. The cloning of the WSN (H1N1) *PB1* gene into a pCAGGS vector has been described previously [28]. All constructs were validated by sequencing. 

### 2.3. Antibodies

The mouse monoclonal antibodies (mAbs) against the PB2, PB1, PA, and NP proteins of IAV were generated in our laboratory [29]. The following primary antibodies were obtained from commercial sources: the rabbit anti-HACD3 polyclonal antibody (pAb) (28572-1-AP; Proteintech, Wuhan, China), rabbit anti-SQSTM1/p62 pAb (18420-1-AP; Proteintech), rabbit anti-Myc pAb (A00172; Genscript, Nanjing, China), mouse anti-Myc mAb (A00704; Genscript), rabbit anti-GST pAb (A00097; Genscript), rabbit anti-V5 pAb (AB3792; Sigma-Aldrich, Saint Louis, MO, USA), mouse anti-V5 mAb (V8012; Sigma-Aldrich), mouse anti-GAPDH mAb (60004-1-Ig; Proteintech), rabbit anti-HA pAb (11692-T62; Sino Biological, Beijing, China), rabbit anti-NA pAb (GTX125974; Genetex, Irvine, CA, USA), rabbit anti-M1 pAb (GTX125928; Genetex), rabbit anti-NS1 pAb (GTX125990; Genetex). The secondary antibodies, DyLight 800 goat anti-mouse IgG (H+L) (072-07-18-06; KPL, Gaithersburg, MD, USA) and DyLight 800 goat anti-rabbit IgG (H+L) (072-07-15-06; KPL), were used for Western blotting. The secondary antibodies, Alexa Fluor 488 goat anti-rabbit IgG (H+L) (A-11034; Invitrogen, Carlsbad, CA, USA) and Alexa Fluor 633 goat anti-mouse IgG (H+L) (A-21052; Invitrogen), were used for confocal microscopy.

### 2.4. siRNA Transfection and Virus Infection

Scrambled siRNA or siRNA targeting *HACD3* (5′-GGCGCAGAAUGCUUUGAAATT-3′; 5′-UUUCAAAGCAUUCUGCGCCTT-3′) was transfected into A549 cells by the use of Lipofectamine RNAiMAX (Invitrogen). At 48 h post-transfection, quantitative reverse-transcription PCR (RT-qPCR) was performed to determine the knockdown efficiency of *HACD3*. siRNA-treated A549 cells were infected with the WSN (H1N1) virus [multiplicity of infection (MOI) = 0.01], and at 24 and 48 h post-infection (p.i.), the supernatants were titrated for infectious viruses by means of plaque assays on MDCK cells. In addition, siRNA-treated A549 cells were also infected with the WSN (H1N1) virus at an MOI of 5. Whole-cell lysates collected at the indicated time points were analyzed by Western blotting with a rabbit anti-HACD3 pAb and antibodies against viral proteins.

### 2.5. Plasmid Transfection and Virus Infection

A549 cells were transfected with plasmids expressing HACD3 or empty pCAGGS vectors using Lipofectamine 2000 reagent (Invitrogen). Twenty-four hours later, the transfected cells were infected with the WSN (H1N1) virus (MOI = 0.01 or 5). At 24 and 48 h (MOI = 0.01), or 12 and 24 h (MOI = 5) p.i., supernatants were titrated for infectious viruses by means of plaque assays on MDCK cells.

HEK293T cells were transfected with scrambled siRNA or *HACD3* siRNA, and at 24 h post-transfection, the cells were re-transfected with constructs expressing WSN (H1N1) PB1 or empty vectors. Forty-eight hours later, the levels of *PB1* mRNA were determined by means of RT-qPCR. In addition, the siRNA-treated cells were transfected with plasmids expressing WSN (H1N1) PB1 or empty vectors. At 36 h post-transfection, the cells were treated with the indicated inhibitors for 4 h and then analyzed by Western blotting with a rabbit anti-HACD3 pAb and a mouse anti-PB1 mAb.

### 2.6. RT-qPCR Assay 

RT-qPCR assays were conducted by using TB Green Premix Ex Taq II (Tli RNaseH Plus) (TaKaRa, Kusatsu, Shiga, Japan). Relative RNA quantities were analyzed as described previously [27]. To ensure specific detection, dissociation curve analysis was performed after each assay. 

### 2.7. Co-Immunoprecipitation (Co-IP) Assay

To investigate interactions between proteins, the indicated plasmids were transfected into HEK293T cells with Lipofectamine 2000. Forty-eight hours later, the transfected cells were washed with ice-cold PBS, and cell lysates were prepared with IP lysis buffer (Pierce, Rockford, IL, USA) supplemented with a protease inhibitor (Roche, Mannheim, Germany) and PMSF (10 mM; Beyotime, Shanghai, China). After centrifugation for 10 min at 12,000 rpm, the supernatants were incubated with the corresponding primary antibodies and a protein G-Agarose immunoprecipitation reagent (Roche). The mixture was rocked at 4 °C for 6 h, followed by four rounds of washing with IP buffer [1% (*v*/*v*) NP-40, 50 mmol/L Tris-HCl, 50 mmol/L EDTA, 150 mmol/L NaCl, pH 7.4]. The bound proteins were resolved by SDS-PAGE, transferred to nitrocellulose membranes, and subjected to Western blotting with the indicated antibodies.

### 2.8. Confocal Assay

A549 cells were uninfected or infected with the WSN (H1N1) virus (MOI = 5) at 4 °C for 60 min, incubated at 37 °C for 8 h, fixed with 4% PFA for 30 min, and permeabilized with 0.1% (*v*/*v*) Triton X-100 in PBS for 15 min. The permeabilized cells were blocked with 5% BSA in PBS for 1 h, incubated with the indicated primary antibodies, mouse anti-PB1 mAb and rabbit anti-HACD3 pAb, at 4 °C overnight, and stained with secondary antibodies conjugated to Alexa Fluor 488 and Alexa Fluor 633. Nuclei were counterstained with DAPI. Imaging of the cells was carried out using a ZEISS laser-scanning confocal microscope.

### 2.9. Western Blotting

Protein samples resolved by SDS-PAGE were transferred to nitrocellulose membranes (GE Healthcare, Pittsburgh, PA, USA). The membranes were blocked with 5% skim milk at room temperature (RT) for 1 h, followed by incubation with the appropriately diluted primary antibodies at 4 °C for 8 h. After washing three times with PBST, the membranes were incubated with the corresponding secondary antibodies diluted in PBS at RT for 1 h and then washed three times with PBST. Finally, the membranes were scanned with an Odyssey CLX infrared imaging system (Li-Cor BioScience, Lincoln, NE, USA).

### 2.10. Dual-Luciferase Reporter Assay

The vRNP complex activity of IAV was analyzed by performing a dual luciferase reporter assay as described previously [30]. In brief, HEK293T cells were transfected with scrambled siRNA or *HACD3* siRNA for 24 h or were transfected with plasmids expressing V5-tagged HACD3 or empty pCAGGS vectors for 24 h. The cells were then further transfected with plasmids expressing the four RNP complex proteins (PB2, PB1, PA, and NP) of the WSN (H1N1) virus, pHH21-SC09NS F-Luc, and pRL-TK. Forty-eight hours later, cells were lysed with the lysis buffer of the dual-luciferase reporter assay system (Promega, Madison, WI, USA), and the luciferase activities were detected on a GloMax 96 microplate luminometer (Promega).

### 2.11. Cell Viability Assay

Cell viability was examined as described previously [28]. In brief, cells seeded in opaque-walled 96-well plates were subjected to the indicated treatment, followed by the addition of 100 µL of CellTiter-Glo reagent into each well. The plate was gently rocked at RT for 10 min on a shaker, and the luminescence was then detected on the GloMax 96 Microplate Luminometer.

### 2.12. Plaque Assay

Titers of the WSN (H1N1) virus were determined by plaque assays on MDCK cells. In brief, MDCK cells were grown to 95% confluency in 12-well plates and were then infected with virus-containing samples at 37 °C for 1 h. After removing the inoculum, the cells were washed with 1 × MEM (Life Technologies, Grand Island, NY, USA) and overlaid with 1% SeaPlaque agarose (Lonza, Rockland, ME, USA) in 1 × MEM containing 0.3% bovine serum albumin (BSA, Sigma-Aldrich) and 0.5 µg/mL TPCK-treated trypsin (Worthington, Lakewood, NJ, USA). After the agarose was solidified at RT, the cells were incubated at 37 °C for 48 h for plaque formation, and the plaques were then counted.

## 3. Results

### 3.1. siRNA Knockdown of HACD3 Suppresses the Replication of IAV

**Figure 1:**
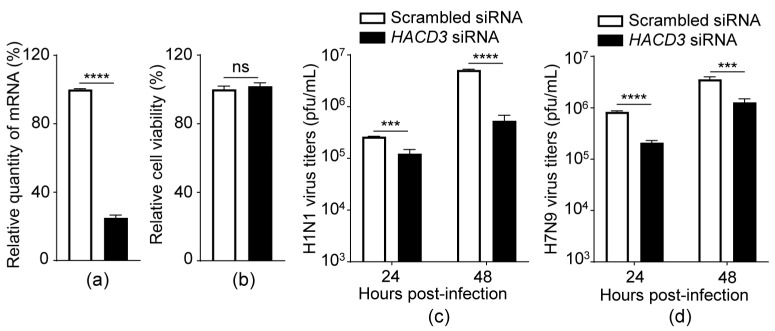
siRNA knockdown of HACD3 suppresses the replication of IAV. (**a**) A549 cells were transfected with scrambled siRNA or *HACD3* siRNA (30 pmol) for 48 h and the mRNA level of *HACD3* was detected by RT-qPCR (*n* = 3). (**b**) Viability of A549 cells treated with scrambled siRNA or *HACD3* siRNA (30 pmol) was assessed by using a CellTiter-Glo assay (*n* = 3). (**c**,**d**) A549 cells treated with scrambled siRNA or *HACD3* siRNA (30 pmol) for 24 h were infected with WSN (H1N1) (MOI = 0.01) (**c**) or AH13 (H7N9) (MOI = 0.01) (**d**). Virus titers in the supernatants were determined by plaque assays on MDCK cells (*n* = 3) at 24 and 48 h p.i. ns, not significant; ***, *p* < 0.001; ****, *p* < 0.0001. Data are representative of three independent experiments (**c**,**d**).

Given the emerging role of HACD3 in the replication of HCV, we then asked whether HACD3 is also involved in the life cycle of IAV. The effect of HACD3 on IAV replication was first assessed when the expression of HACD3 was silenced in A549 cells that were transfected with scrambled siRNA or siRNA targeting *HACD3*. At 24 h post-transfection, the WSN (H1N1) virus (MOI = 0.01) was used to infect the siRNA-treated cells, and the virus growth titers in the culture supernatants were titrated by plaque assay at 24 and 48 h p.i. The level of *HACD3* mRNA was significantly downregulated in cells treated with *HACD3* siRNA compared with scrambled siRNA (Figure 1a), without producing adverse effects on cell viability (Figure 1b). The silencing of HACD3 expression led to 2.1- and 9.6-fold reductions of progeny virus titers compared with those of scrambled siRNA-treated cells at 24 and 48 h p.i., respectively (Figure 1c). Similarly, HACD3 knockdown also dramatically declined the yield of the A/Anhui/1/2013 (AH13, H7N9) virus (Figure 1d). These results indicate that endogenous HACD3 is required for the efficient replication of IAV.

### 3.2. Overexpression of HACD3 Promotes the Replication of IAV

**Figure 2:**
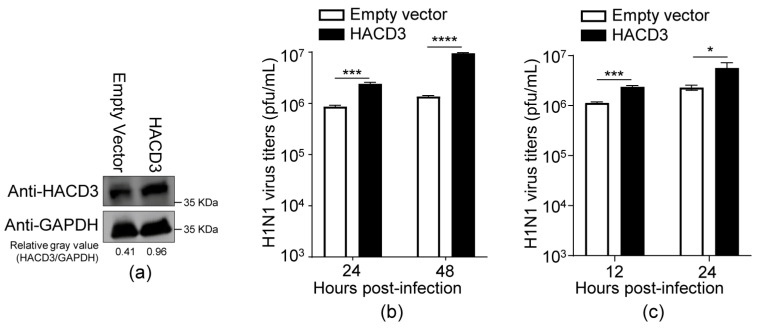
Overexpression of HACD3 promotes the replication of IAV. (**a**) A549 cells grown in 12-well plates were transfected with HACD3-expressing plasmids or empty vectors (0.5 µg) for 24 h, and the expression of HACD3 was detected by Western blotting with a rabbit anti-HACD3 pAb. (**b**,**c**) A549 cells were transfected as in (**a**), and then infected with the WSN (H1N1) virus at an MOI of 0.01 (**b**) or 5 (**c**). Virus titers in the supernatants were determined by plaque assays on MDCK cells (*n* = 3) at 24 and 48 h p.i. (**b**) or 12 and 24 h p.i. (**c**). *, *p* < 0.05; ***, *p* < 0.001; ****, *p* < 0.0001. Data are representative of three independent experiments (**b**,**c**).

To further investigate the role of HACD3 in the replication of IAV, A549 cells were transfected with the empty pCAGGS vectors or HACD3-expressing plasmids to transiently overexpress HACD3 (Figure 2a). At 24 h post-transfection, the A549 cells were infected with the WSN (H1N1) virus (MOI = 0.01). As shown in Figure 2b, the transient overexpression of HACD3 in A549 cells resulted in 2.8- and 7.0-fold increases of virus growth titers at 24 and 48 h p.i., respectively. In addition, we also infected the transfected A549 cells with the WSN (H1N1) virus at an MOI of 5 and observed 2.1- and 2.5-fold increases of virus growth titers in HACD3-overexpressing cells at 12 and 24 h p.i., respectively (Figure 2c). Together, these results validate that HACD3 is a positive host cellular factor to support the replication of IAV.

### 3.3. HACD3 Downregulation Suppresses the Expression of the IAV PB1 Protein

**Figure 3:**
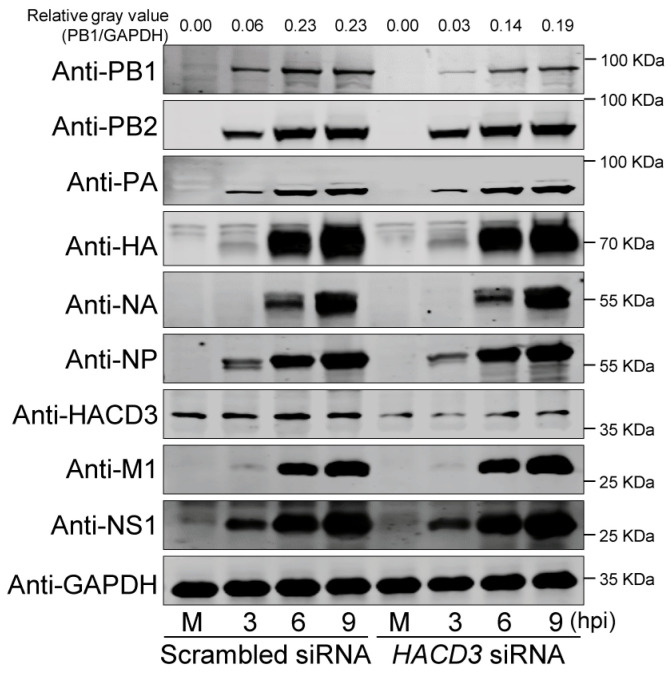
Silencing HACD3 expression reduces the level of PB1 protein in IAV-infected cells. A549 cells were treated with scrambled siRNA or *HACD3* siRNA (30 pmol) and then infected with the WSN (H1N1) virus at an MOI of 5. Whole-cell lysates were prepared at the indicated timepoints and Western blotted with a mouse anti-PB2, anti-PB1, anti-PA, or anti-NP mAb, or a rabbit anti-HA, anti-NA, anti-M1, anti-NS1, or anti-HACD3 pAb. M, Mock. The ratios of gray values were analyzed by ImageJ 1.51n.

To investigate whether HACD3 affects the replication of IAV by modulating the expression of viral proteins, we infected scrambled siRNA- or *HACD3* siRNA-treated A549 cells with the WSN (H1N1) virus (MOI = 5), and whole-cell lysates were Western blotted at 3, 6, and 9 h p.i. to detect the levels of viral proteins. Interestingly, we found that HACD3 silencing remarkably decreased the expression level of viral PB1 protein while having no effect on the expression of any other viral proteins (Figure 3). These results demonstrate that HACD3 is most likely involved in the replication of IAV by specifically regulating viral PB1 expression. 

### 3.4. HACD3 Silencing Promotes PB1 Degradation through the Lysosome Pathway

**Figure 4:**
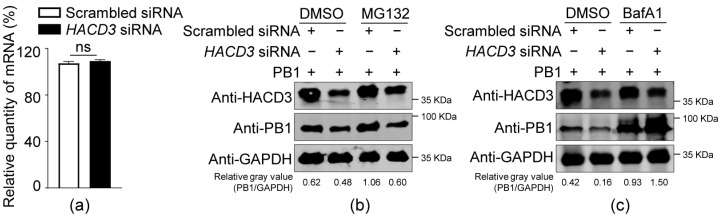
HACD3 silencing promotes PB1 degradation through the lysosome pathway. (**a**) HEK293T cells were transfected with scrambled siRNA or *HACD3* siRNA (30 pmol) for 24 h, and then further transfected with PB1-expressing plasmids or empty vectors (1.0 µg). Forty-eight hours later, the levels of *PB1* mRNA were determined by RT-qPCR (*n* = 3). ns, not significant. (**b**,**c**) HEK293T cells were transfected with scrambled siRNA or *HACD3* siRNA (30 pmol) for 24 h, and then further transfected with PB1 protein-expressing plasmids (1.0 µg). Thirty-six hours later, the cells were treated with MG132 (**b**) or BafA1 (**c**) for 4 h, and then Western blotted with a rabbit anti-HACD3 pAb and a mouse anti-PB1 mAb. The ratios of gray values were analyzed by ImageJ 1.51n.

To explore the mechanism of impaired expression of PB1 protein upon HACD3 silencing, scrambled siRNA- or *HACD3* siRNA-treated HEK293T cells were transfected with PB1-expressing plasmids. RT-qPCR analysis showed that the levels of *PB1* mRNA were comparable between HACD3-silenced cells and scrambled siRNA-treated cells (Figure 4a), indicating that the role of HACD3 in PB1 expression is not achieved by affecting the transcription of *PB1* mRNA. We then determined how HACD3 affects the expression of PB1 at the protein level. To this end, scrambled siRNA- or *HACD3* siRNA-treated HEK293T cells were transfected with PB1-expressing plasmids. At 36 h post-transfection, the cells were treated with proteasome inhibitor MG132 or autophagy inhibitor Bafilomycin A1 (BafA1). Western blotting of whole-cell lysates showed that the BafA1 treatment counteracted the destabilizing effect of HACD3 silencing on PB1, a phenomenon that was not observed in MG132 treatment (Figure 4b,c). These results indicate that the siRNA-mediated HACD3 knockdown leads to PB1 protein degradation through the lysosome pathway. 

### 3.5. HACD3 Overexpression Enhances the Expression of PB1 Protein

**Figure 5:**
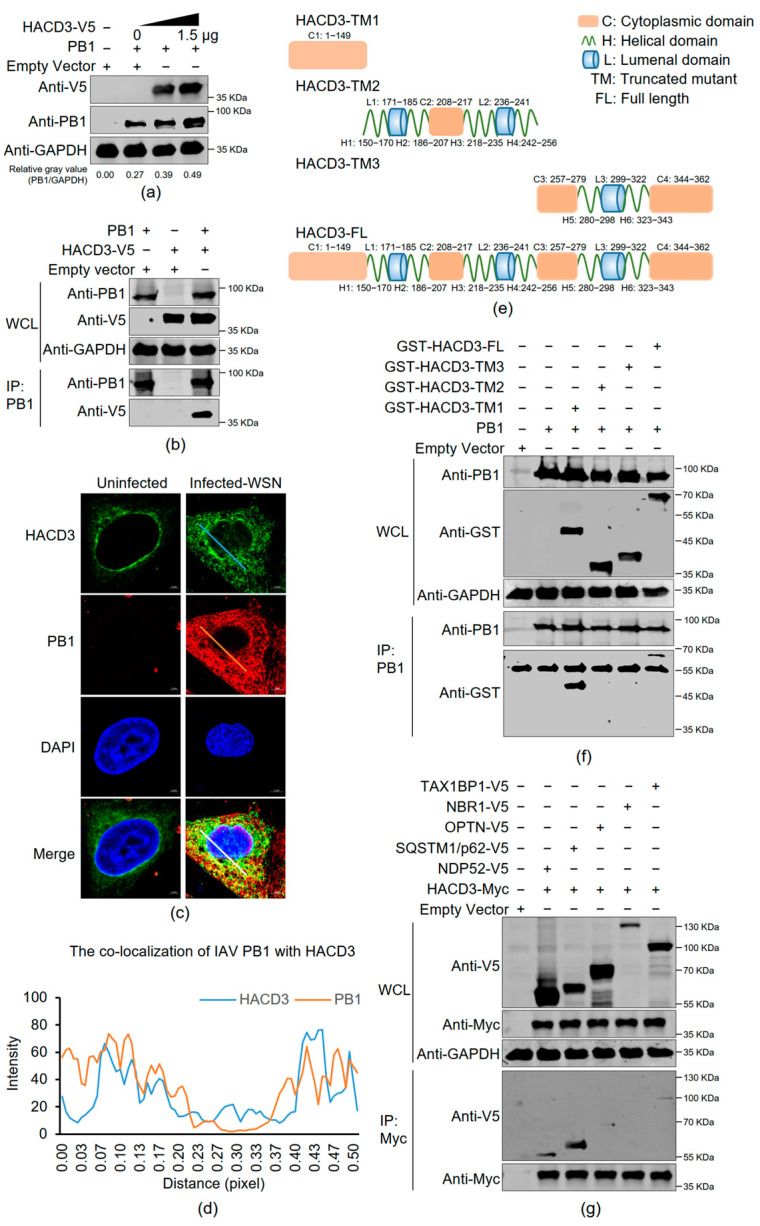
HACD3 interacts with IAV PB1 and SQSTM1/p62. (**a**) HEK293T cells were transfected with plasmids (1.0 µg) to express WSN (H1N1) PB1 and gradually increasing quantities of V5-tagged HACD3. At 48 h post-transfection, cell lysates were subjected to Western blotting with a rabbit anti-V5 pAb and a mouse anti-PB1 mAb. (**b**) HEK293T cells were transfected with the indicated combinations of plasmids (2.0 µg/each) for 48 h. Cell lysates were incubated with a mouse anti-PB1 mAb for immunoprecipitation, and the immunoprecipitated proteins were Western blotted with a rabbit anti-V5 pAb and a mouse anti-PB1 mAb. (**c**) A549 cells were uninfected or infected with the WSN (H1N1) virus (MOI = 5) for 8 h, and then examined by confocal microscopy to visualize the co-localization of viral PB1 protein and HACD3. These cells were fixed and stained with a mouse anti-PB1 mAb and a rabbit anti-HACD3 pAb, followed by incubation with Alexa Fluor 633 goat anti-mouse IgG (H+L) (red) and Alexa Fluor 488 goat anti-rabbit IgG (H+L) (green). The nuclei were stained with DAPI. (**d**) The co-localization of viral PB1 with HACD3 as in (**c**) was analyzed by ImageJ 1.51n. (**e**) Schematic presentation of full-length HACD3 and its truncated mutants based on the HACD3 structure in AlphaFold Protein Structure Database (AFDB accession: AF-Q9P035-F1). (**f**) HEK293T cells were transfected with plasmids to express WSN (H1N1) PB1 and GST-tagged full-length or truncated HACD3 (2.0 µg/each). Forty-eight hours later, cell lysates were incubated with a mouse anti-PB1 mAb for immunoprecipitation, and the immunoprecipitated proteins were Western blotted with a rabbit anti-GST pAb and a mouse anti-PB1 mAb. (**g**) HEK293T cells were transfected with plasmids to express HACD3-Myc and individual V5-tagged autophagy receptors or empty vectors (2.0 µg). Forty-eight hours later, cell lysates were incubated with a mouse anti-Myc mAb for immunoprecipitation, and the immunoprecipitated proteins were Western blotted with a rabbit anti-V5 pAb and anti-Myc pAb. The ratios of gray values were analyzed by ImageJ 1.51n.

To further determine the effect of HACD3 on the expression of IAV PB1, HEK293T cells were transfected individually with PB1-expressing plasmids or in combination with a gradually increasing quantity of HACD3-V5-expressing plasmids. At 48 h post-transfection, cell lysates were Western blotted to detect the expression level of PB1. We found that the level of PB1 expression was gradually enhanced along with the gradient increase of HACD3 expression (Figure 5a), indicating that HACD3 functions to facilitate the expression of IAV PB1 protein. 

### 3.6. HACD3 Interacts with IAV PB1 and SQSTM1/p62

To define the relationship between HACD3 and the IAV PB1 protein, HEK293T cells were transfected individually or in combination with plasmids to express V5-tagged HACD3 and WSN (H1N1) PB1. The Co-IP experiment showed that HACD3 interacted with PB1 (Figure 5b). Then, the localization of PB1 and HACD3 was monitored in A549 cells infected with the WSN (H1N1) virus (MOI = 5). We found that PB1 clearly co-localized with HACD3 in the cytoplasm at 8 h p.i. (Figure 5c,d). Next, to further map the specific domain of HACD3 that mediates the interaction with PB1, we generated a set of GST-tagged constructs expressing the full-length form (amino acids 1–362) or truncated mutants (amino acids 1–149, 150–256, or 257–362) of HACD3 (Figure 5e). Co-IP experiments in HEK293T cells showed that the amino acids 1–149 harboring the first cytoplasmic domain of HACD3 were responsible for the interaction with IAV PB1 (Figure 5f). In addition, we also performed a Co-IP experiment in HEK293T cells that were transfected with plasmids expressing HACD3 and individual autophagy receptors, i.e., V5-tagged NDP52, SQSTM1/p62, OPTN, NBR1, and TAX1BP1. We found that HACD3 robustly interacted with SQSTM1/p62 and bound weakly to NDP52, but had no interaction with other autophagy receptors (Figure 5g). 

### 3.7. HACD3 Competes with SQSTM1/p62 in the Interaction with PB1

**Figure 6:**
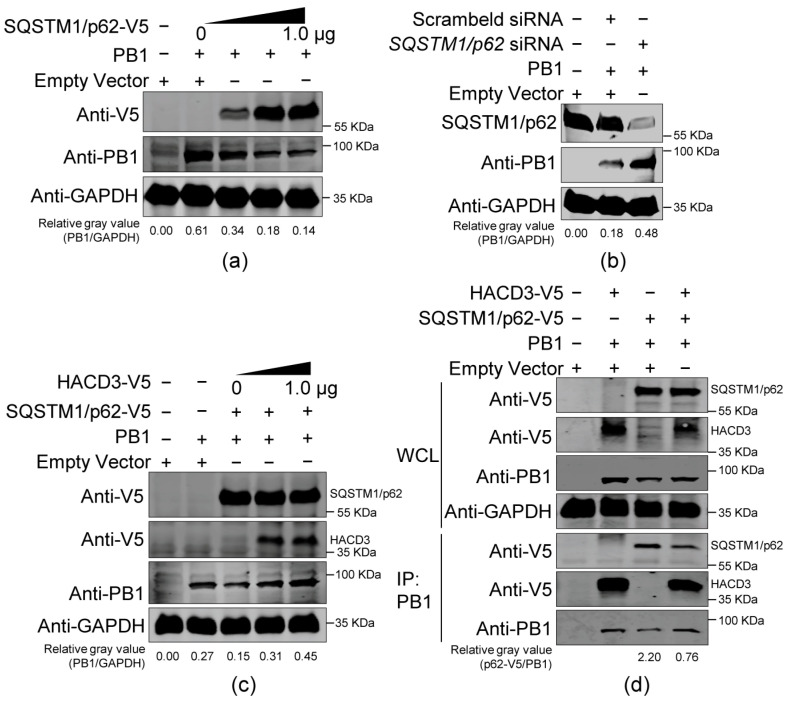
HACD3 competes with SQSTM1/p62 in the interaction with IAV PB1. (**a**) HEK293T cells were transfected with plasmids (1.0 µg) to express WSN (H1N1) PB1 and a gradually increasing amount of V5-tagged SQSTM1/p62. At 48 h post-transfection, cell lysates were subjected to Western blotting with a rabbit anti-V5 pAb and a mouse anti-PB1 mAb. (**b**) HEK293T cells were transfected with *SQSTM1/p62* siRNA or scrambled siRNA (30 pmol), and at 24 h post-transfection, the cells were re-transfected with plasmids to express WSN (H1N1) PB1 or empty vectors (1.0 µg). Thirty-six hours later, cell lysates were subjected to Western blotting with a rabbit anti-SQSTM1/p62 pAb and a mouse anti-PB1 mAb. (**c**) HEK293T cells were transfected with the indicated combinations of plasmids expressing WSN (H1N1) PB1 (1.0 µg), SQSTM1/p62-V5 (1.0 µg), and gradually increasing amounts of V5-tagged HACD3. At 48 h post-transfection, cell lysates were subjected to Western blotting with a rabbit anti-V5 pAb and a mouse anti-PB1 mAb. (**d**) The indicated combinations of plasmids (2.0 µg/each) were transfected into HEK293T cells to express WSN (H1N1) PB1, HACD3-V5, and SQSTM1/p62-V5. At 48 h post-transfection, cell lysates were incubated with a mouse anti-PB1 mAb for immunoprecipitation, and the immunoprecipitated proteins were Western blotted with a rabbit anti-V5 pAb and a mouse anti-PB1 mAb. The ratios of gray values were analyzed by ImageJ 1.51n.

To determine the effect of SQSTM1/p62 on the expression of IAV PB1, HEK293T cells were transfected with PB1-expressing plasmids alone or in combination with a gradually increasing quantity of SQSTM1/p62-V5-expressing plasmids. At 48 h post-transfection, cell lysates were Western blotted to detect the expression level of PB1. We found that the level of PB1 expression was gradually weakened along with the gradient increase in SQSTM1/p62 expression (Figure 6a), indicating that SQSTM1/p62 acts to suppress the expression of IAV PB1 protein. To validate this finding, HEK293T cells were transfected with *SQSTM1/p62* siRNA or scrambled siRNA, and at 24 h post-transfection, the cells were re-transfected with plasmids to express PB1 or empty vectors. Thirty-six hours later, cell lysates were Western blotted for the detection of PB1. We found that the expression level of PB1 was dramatically enhanced in *SQSTM1/p62* siRNA- versus scrambled siRNA-transfected cells (Figure 6b), thereby confirming the negative regulating role of HACD3 on the expression of IAV PB1. Next, HEK293T cells were transfected with the indicated combinations of plasmids to express PB1, SQSTM1/p62-V5, and gradually increasing quantities of HACD3-V5. At 48 h post-transfection, cell lysates were Western blotted to detect the expression level of PB1. We found that the expression of PB1 protein was dramatically impaired when SQSTM1/p62 was co-expressed. However, the suppressing effect of SQSTM1/p62 on PB1 expression was antagonized along with the gradient increase in HACD3 expression (Figure 6c). We also attempted to investigate the relationship among HACD3, PB1, and SQSTM1/p62. Co-IP experiments were performed in HEK293T cells transfected with different combinations of plasmids expressing WSN (H1N1) PB1, HACD3-V5, and SQSTM1/p62-V5. As shown in Figure 6d, PB1 interacted with both HACD3 and SQSTM1/p62 when they were co-transfected. Notably, the interaction between PB1 and SQSTM1/p62 was dramatically impaired when HACD3 was co-expressed. These results indicate that HACD3 competes with SQSTM1/p62 in the interaction with PB1, thereby protecting the PB1 protein from recognition by the autophagy receptor and subsequent autophagic degradation. 

### 3.8. HACD3 Positively Regulates the vRNP Complex Activity of IAV

**Figure 7:**
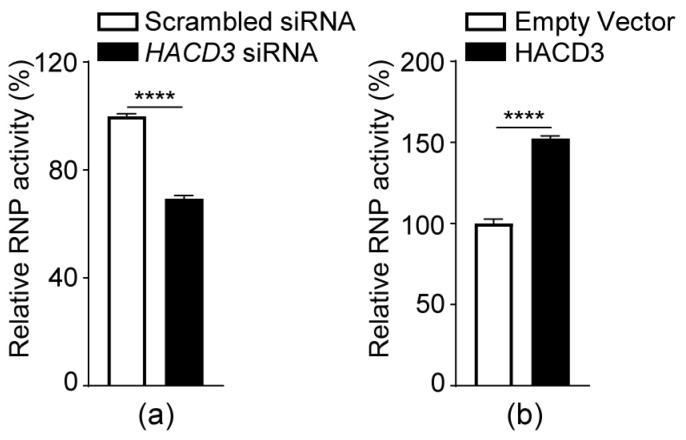
HACD3 positively regulates the vRNP complex activity of IAV. HEK293T cells were treated with scrambled siRNA or *HACD3* siRNA (30 pmol) for 24 h (**a**) or were transfected with plasmids expressing HACD3 or empty vectors (1.0 µg) for 24 h (**b**). The cells were then further transfected with the four protein expression constructs (PB2, PB1, PA, and NP; 0.5 µg each) of the vRNP complex of the WSN (H1N1) virus, pHH21-SC09NS F-Luc (0.1 µg), and pRL-TK (0.02 µg). Forty-eight hours later, cell lysates were prepared with a dual-luciferase reporter assay system and were measured for luciferase activities on a GloMax 96 microplate luminometer (*n* = 3). ****, *p* < 0.0001. Data are representative of three independent experiments.

Given that HACD3 functions to prevent PB1 from autophagic degradation, we speculate that HACD3 may be involved in regulating the vRNP complex activity of IAV. To test this hypothesis, HEK293T cells were transfected with scrambled siRNA or *HACD3* siRNA for 24 h or were transfected with plasmids to express HACD3 or empty pCAGGS vectors for 24 h. The cells were then further transfected with plasmids expressing PB2, PB1, PA, and NP of the WSN (H1N1) virus, together with luciferase reporter constructs. Forty-eight hours later, cell lysates were subjected to luciferase assays to reveal vRNP activity. As shown in Figure 7a, vRNP activity was reduced by 30% in *HACD3* siRNA- versus scrambled siRNA-treated cells, whereas activity was increased by approximately 50% when the expression of HACD3 was exogenously complemented (Figure 7b). These results indicate that the presence of HACD3 is important for the activity of the vRNP complex of IAV.

## 4. Discussion

IAV constitutes a global challenge and threat to human health and the animal industry. Due to the tough features of IAV, e.g., co-existence of numerous subtypes, persistent evolution, and wide host species, it is almost impossible to completely eradicate IAV in nature [31,32]. Therefore, it is particularly important to discover novel therapeutic targets for the development of effective countermeasures, which absolutely relies on a profound understanding of how the virus-host interplay drives the replication of IAV in host cells. A number of host cellular factors have been discovered to participate in different stages of the replication cycle of IAV, e.g., endocytosis, nuclear import of the vRNP complex, transcription and replication of the viral genome, and assembly and budding [33,34,35]. However, relatively few interacting host cellular factors have been revealed to interact with PB1, the core subunit of the viral polymerase complex. 

HACD3 possesses 3-hydroxyacyl-CoA dehydratase activity, which is involved in the fatty acid biosynthetic process [22,23]. To date, HACD3 has only been implicated in the replication of HCV. It interacts with the NS5A protein of HCV and also uses an N-terminal FxxW motif to bind Hsp90, which is important for the genomic RNA replication and particle production of HCV [26]. In the present study, we defined HACD3 as a novel host factor that positively regulates the replication of IAV. siRNA-mediated downregulation of HACD3 expression suppressed the replication of the WSN (H1N1) and AH13 (H7N9) viruses, whereas overexpression of HACD3 by transient transfection of HACD3-expressing plasmids promoted WSN (H1N1) virus replication. Interestingly, the downregulation of HACD3 expression by siRNA treatment specifically reduced the expression level of viral PB1 protein at 3, 6, and 9 h p.i., but had no effect on the expression of other viral proteins. In addition to being a component of the vRNP complex of IAV, PB1 can be present as a free molecule when it is first translated in the cytoplasm. Most likely, the siRNA-mediated silencing of HACD3 led to the reduction of the levels of free PB1 rather than the PB1 within the vRNP complex. As a result, it appeared that the *HACD3* siRNA treatment decreased the level of PB1 but not other viral proteins. Moreover, we found that the co-expression of HACD3 with PB1 in HEK293T cells could increase the level of PB1 expression in a dose-dependent manner, further demonstrating that HACD3 plays a promoting role in the regulation of the expression level of IAV PB1 protein.

Subsequently, we explored the underlying mechanism by which HACD3 promoted the expression of the PB1 protein. By the transfection of PB1-expressing plasmids in *HACD3* siRNA- or scrambled siRNA-treated HEK293T cells, we revealed that the downregulation of HACD3 expression had no effect on the level of *PB1* mRNA, indicating the promoting role of HACD3 on PB1 protein expression is not achieved at the transcription level. To date, HACD3 has never been implicated to play a role in the autophagy pathway. By examining the level of PB1 under the treatment of proteasome inhibitor MG132 and autophagy inhibitor BafA1, we found that only BafA1 could rescue the expression of the PB1 protein in HACD3-silenced cells, indicating that the downregulation of HACD3 expression promotes the degradation of the PB1 protein through the autophagy lysosomal pathway. In an effort to identify the selective autophagy receptor that functions in HACD3-mediated regulation of PB1 expression, we found that HACD3 interacted with both PB1 and SQSTM1/p62. Importantly, HACD3 competed with SQSTM1/p62 to interact with PB1, thereby preventing PB1 from entering the autophagic degradation pathway. Consistently, HACD3 positively regulated vRNP complex activity, thus facilitating the replication of IAV.

## 5. Conclusions

**Figure 8:**
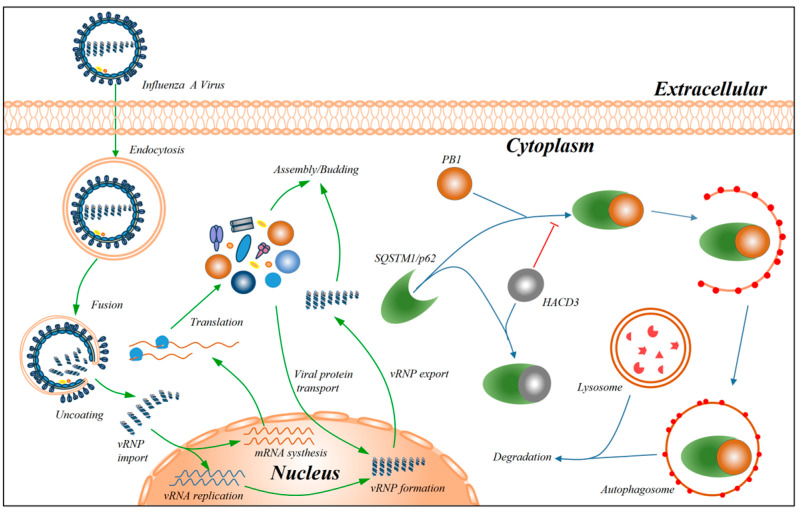
Diagram showing the role of HACD3 in facilitating the replication of IAV. HACD3 interacted with both PB1 and SQSTM1/p62, and most importantly, HACD3 competed with SQSTM1/p62 to interact with PB1, thereby preventing PB1 from entering the autophagic degradation pathway. As a result, HACD3 positively regulated vRNP complex activity and facilitated the replication of IAV.

In summary, our results demonstrated that HACD3, as a new host factor that positively regulates IAV replication, protects the PB1 protein from autophagic degradation by competing with autophagy receptor SQSTM1/p62 for binding with PB1 (Figure 8). 

## Data Availability

The data presented in the study are included in the article, further inquiries can be directed to the corresponding authors.
